# Synthesis of 5-arylidine amino-1,3,4-thiadiazol-2-[(*N*-substituted benzyol)]sulphonamides endowed with potent antioxidants and anticancer activity induces growth inhibition in HEK293, BT474 and NCI-H226 cells

**DOI:** 10.1007/s00044-013-0890-z

**Published:** 2013-12-27

**Authors:** Mahavir Chhajed, Anil Kumar Shrivastava, Vijay Taile

**Affiliations:** 1 Department of Pharmaceutical Chemistry, Suresh Gyan Vihar University, Mahal Jagat Pura, Jaipur, India; 2 Nandini Nagar Mahavidyalaya College of Pharmacy, Nawabganj, Gonda, Uttar Pradesh India; 3 Department of Chemistry, RTM Nagpur University, Nagpur, India

**Keywords:** 1,3,4-Thiadiazole, Antimitotic, Antioxidants, Cytotoxicity, MTT assay

## Abstract

**Abstract:**

A series of imines 5-amino-1,3,4-thiadiazol-2-[(*N*-substituted benzyol)]sulphonamide derivatives were synthesized from various aromatic aldehydes and substituted with benzoyl acetazolamides under different reaction conditions and were evaluated for their antioxidant and free radical scavenging, antimitotic activity by *Allium cepa* meristem root model and cytotoxicity activity against HEK 293 (human epidermal kidney cell line), BT474 (breast cancer cell line) and NCI-H226 (lung cancer cell line) by MTT assay. Some of the synthesized compounds showed moderately potent cytotoxicity compared to indisulam.

**Graphical abstract:**

A series of imines 5-amino-1,3,4-thiadiazol-2-[(*N*-substituted benzyol)]sulphonamide derivatives (**9a**–**j**); 5-amino-1,3,4-thiadiazol-2-[*N*-(substituted benzoyl)]sulphonamide (**4a**–**g**); 5-(4-acetamido phenyl sulphonamido)-1,3,4-thiadiazol-2-[*N*-(substituted benzoyl)]sulphonamide (**6a**–**g**); and 5-(4-amino phenyl sulphonamido)-1,3,4-thiadiazol-2-[*N*-(substituted benzoyl)]sulphonamide (**7a**–**g**) were synthesized from acetazolamide and were investigated for the in vitro anticancer by MTT assay, free radical scavenging and antimitotic activity by *Allium cepa* root meristem model. Experimental observations indicate that synthesized compounds were moderately potent anticancer agents. 
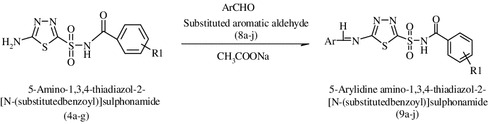

## Introduction

The rapid spread of cancer has sparked an intense worldwide search for new compounds, which may be used in designing anticancer drugs. The search of more effective anticancer agent has focused to a large extent on the design of molecules capable of recognizing and binding to target DNA base sequences. Development of anticancer drugs with fewer or no side effects is important for the treatment for cancer. The search for such potential anticancer drugs has led to the discovery of synthetic small molecules with anti-carcinogenic activity and limited harmful side effects particularly with respect to the immune system. Research in this area is expanding rapidly, and some promising leads have emerged. Heterocyclic moieties can be found in a large number of compounds, which display biological activity. The biological activity of the compounds is mainly dependent on their molecular structures (Salimon *et al*., 2010). A vast number of 1,3,4-thiadiazoles have been reported as potential pharmacologically active compounds with antimicrobial (Patil and Biradar, 2001; Zamani *et al*., 2004; Sharma *et al*., 2006), antiviral (Pandey *et al*., 2004), antitubercular (Oruc *et al*., 2004; Desai *et al*., 1984), anticonvulsant (Shrivastava *et al*., 1999; Kumar *et al*., 2003; Gupta *et al*., 2008; Stillings *et al*., 1986; Jatav *et al*., 2008), CNS depressant (Jatav *et al*., 2008), hypoglycaemic (Hanna *et al*., 1995; Pattan *et al*., 2009), anti-inflammatory (Sharma *et al*., 2008; Varandas *et al*., 2005) and anticancer (Noolvi *et al*., 2011; Kumar *et al*., 2010) properties. At the same time, the 1,3,4-thiadiazole fragment appears in a number of clinically used drugs such as acetazolamide; methazolamide; butazolamide (diuretic); sulfamethiazole (antibacterial); cefazolin, cefazedone (antibiotic); atibeprone (anti-depressant); glybuthiazole, glybuzole (antidiabetic); and tebuthiuron (insecticide) (Wilson and Gisvold, 1991; Abrahum, 2003; Supran *et al*., 2003).

Schiff bases, contain an azomethine group, derived from aromatic aldehydes and aromatic amines, have potential for both chemical and biological activities (Dhar and Taploo, 1982; Pacheco *et al*., 1970). This is due to the presence of carbon–nitrogen double bond having potential receptor-binding ability. Schiff bases are also one of the intensively investigated classes of aromatic and heteroaromatic compounds. This class of compounds showed a variety of applications ranging from anticancer (Sharma *et al*., 1998; Kuzmin *et al*., 2005), antibacterial (More *et al*., 2002; Vaghasiya *et al*., 2004), diuretic (Supran *et al*., 1996), antifungal (Manrao *et al*., 1982, 1995, 2001) and antiparasitic activity (Rathelot *et al*., 2002). They have also medicinal importance and are used in drug design due to their activity against a wide range of organisms (Khan *et al*., 2002; Verma *et al*., 2004). Schiff bases are used as substrates in the preparation of a number of industrially and biologically active compounds via closure, cycloaddition and replacement reactions (Taggi *et al*., 2002).

Sulphonamides are a significant class of compounds in medicinal and pharmaceutical chemistry with several biological applications (Tilles, 2001; Slatore and Tilles, 2004; Brackett *et al*., 2004; Harrison, 1994; Eroglu, 2008).

There are many connections between carbonic anhydrase (CA) and cancer (Supuran, 2008; Supuran and Scozzafava, 2000; Pastorek *et al*., 1994; Pastorekova *et al*., 1997; Chegwidden *et al*., 2001). It is well known that some CA isozymes are predominantly found in cancer cells and are lacking from their normal counterparts (Pastorek *et al*., 1994; Pastorekova *et al*., 1997; Chegwidden *et al*., 2001), and these are two transmembrane isozymes CA IX and CA XII. Isozyme CA XIV was the last one to be discovered among the 15 CA isoforms of this widespread metalloprotein known up to now in human (Supuran *et al*., 2004). Kaunisto *et al*. (2002) and Parkkila *et al*., (2001, 2002) revealed CA XIV distribution in the human body as well as potential physiological/pathological roles. It has been observed that hCA XIV is highly abundant in the brain, kidney, colon, small intestine, urinary bladder, liver and spinal cord (Kaunisto *et al*., 2002; Parkkila *et al*., 2001, 2002; Fujikawa-Adachi *et al*., 1999; Ashida *et al*., 2002). Similar to isozymes CA IX and CA XII, CA XIV is a transmembrane protein with the active site oriented extracellularly, but unlike the first two proteins, isozyme XIV is not associated with tumour cells (Pastorek *et al*., 1994; Kaunisto *et al*., 2002; Parkkila *et al*., 2001, 2002; Ashida *et al*., 2002). Membrane-associated human carbonic anhydrase (hCAs) isozymes IX, XII and XIV (Fujikawa-Adachi *et al*., 1999; Tureci *et al*., 1998) like other hCAs regulate pH and carbon dioxide (CO_2_)–bicarbonate anion (HCO_3_) homoeostasis, through the catalysis of the reversible hydration of CO_2_ to give HCO_3_ and proton (Hþ). The expression level of isozymes hCA IX and XII is elevated in response to hypoxia, and research on the involvement of these isozymes in cancer has progressed considerably in recent years, particularly for hCA IX (Tureci *et al*., 1998; Wykoff *et al*., 2000; Parkkila *et al*., 2000; Svastova *et al*., 2004; Cecchi *et al*., 2005). It has been confirmed that hCA IX is a high-activity CA isozyme responsible for the extracellular acidification (pHe) of the tumour microenvironment. Multiple downstream effects of this reduced pHe are associated with tumour progression and poor prognosis (Parkkila *et al*., 2000; Svastova *et al*., 2004). Aromatic sulphonamide compounds have been shown to reverse the effect of tumour acidification, to inhibit the growth of cancer cells and to suppress tumour invasion mediated by these CAs (Tureci *et al*., 1998; Wykoff *et al*., 2000; Parkkila *et al*., 2000; Svastova *et al*., 2004; Cecchi *et al*., 2005; Brzozowski *et al*., 2010).

Thus, the data from these many physiological studies appear to have identified a CA-mediated, hypoxic tumour-specific pathway. This provides firm grounds for exploring the effects of this class of compounds as a novel approach to discriminate between healthy cells and cancerous cells, specifically targeting hypoxic tissues, an attractive attribute that is lacking in many existing cancer therapies (Minchinton and Tannock 2006; Kamb, 2005).

These findings prompted us to the synthesis of 5-arylidine amino-1,3,4-thiadiazol-2-[(*N*-benzoyl)]sulphonamide derivatives (**9a**–**j**) from carbonic anhydrase inhibitor drug acetazolamide. The synthesized compounds reported previously (Chhajed *et al*., 2007, 2013), such as 5-amino-1,3,4-thiadiazol-2-[*N*-(substituted benzoyl)]sulphonamide (**4a**–**g**), 5-(4-acetamido phenyl sulphonamido)-1,3,4-thiadiazol-2-[*N*-(substituted benzoyl)]sulphonamide (**6a**–**g**), and 5-(4-amino phenyl sulphonamido)-1,3,4-thiadiazol-2-[*N*-(substituted benzoyl)]sulphonamide (**7a**–**g**) from acetazolamide by modified Schotten–Bauman synthesis method, and compounds (**9a**–**j**) reported herein are evaluated for anticancer activity, having better therapeutic index for free radical scavenging, antimitotic activity and in vitro cytotoxic activity by MTT assay for establishing their possible therapeutic value. The synthesized molecules have been characterized by various techniques such as NMR, FTIR and LCMS.

## Results and discussion

### Chemistry

**Scheme 1:**
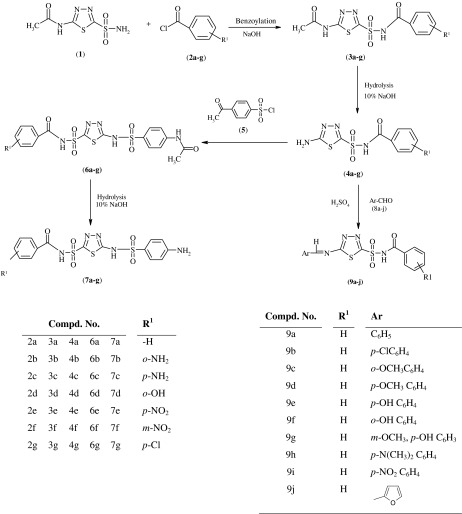
Synthesis of 5-amino-1,3,4-thiadiazol-2-[*N*-(substituted benzoyl)]sulphonamide (**4a**–**g**), 5-(4-acetamido phenyl sulphonamido)-1,3,4-thiadiazole-2-[*N*-(substituted benzoyl)]sulphonamide (**6a**–**g**), 5-(4-amino phenyl sulphonamido)-1,3,4-thiadiazole-2-[*N*-(substituted benzoyl)]sulphonamide (**7a**–**g**), and 5-arylidine amino-1,3,4-thiadiazol-2-[*N*-(substituted benzoyl)]sulphonamide (**9a**–**j**)

5-Amino-1,3,4-thiadiazol-2-[*N*-(substituted benzoyl)]sulphonamides (**4a**–**g**) were prepared by hydrolysis of the benzoylated acetazolamides (**3a**–**g**), which was prepared from the acetazolamide (**1**) by benzoylation with substituted benzoyl chlorides (**2a**–**g**). Compound (**4**) was refluxed with substituted aromatic aldehydes (**8a**–**j**) using concentrated sulphuric acid as a catalyst to obtain the Schiff bases (Scheme 1).

The FT-IR spectra showed stretching at 3,485–3,265 cm^−1^ (sulphonamide N–H), 3,037.4 (Ar C–H), 1,676–1,645 (C=O), 1,625–1,594 cm^−1^ (C=N), 1,517–1,530.9 (Ar C–C), 1,270 cm^−1^ (C–N), 1,177–1,125 cm^−1^ (sulphonamide), 1,128–1,030 cm^−1^ (S=O) and 756–662 cm^−1^ (thiadiazole C–S). The ^1^H-NMR spectra of all compounds indicated expected peaks in the region of 1.249–1.254 *δ* ppm (s, Ar–SO_2_NH), 3.569–4.116 *δ* ppm (s, Schiff base CH=N) and 8.24–8.523 *δ* ppm (s, amide C(=O)N–H), while multiplets of aromatic ring are in the range of 6.6–8.2 *δ* ppm. Thin-layer chromatography (TLC) was run throughout the reaction to optimize the reaction for purity and completion.

### Pharmacological evaluation

#### Antioxidant and free radical scavenging activity

ABTS^**·+**^ radical, lipid peroxidation, DPPH radical, superoxide anion and nitric oxide anion radical scavenging activity has been used as a quick and reliable parameter to assess the in vitro antioxidant activity. Each method relates to the generation of a different radical, acting through a variety of mechanisms and the measurement of a range of end points at a fixed time point or over a range (Miller and Rice-Evans, 1994, 1996). The different concentrations of the synthesized compounds showed antioxidant activities in a dose-dependent manner. Comparative IC_50_ (nM/mL) inhibitory concentrations of synthesized compounds against different free radicals are reported in Table 1. All the tested compounds showed statistically significant (*P* < 0.05) IC_50_ values. Among the tested compounds, (**9c**) is the most potent compound and had lowest IC_50_ (nM/mL) value against DPPH radical, nitric oxide anion and lipid peroxidation, while (**9e**) and **(9f**) showed maximum potency against *ABTS* ^**·+**^ *radical and superoxide anion radical, respectively.* The study also indicates that the compounds (**9c**), (**9d**) and (**9f**) showed the smaller IC_50_ (nM/mL) values even than respective standards, indicating that these compounds are more potent than the standard, and reveals that the electron-donating functional group like –OCH_3_ (**9c** and **9d**) or the functional group like –OH having the ability to bind with free radical (**9f**) is responsible for the potency.

**Table 1:** Comparative IC_50_ inhibitory concentration of synthesized compounds and standards against different free radicals

Compound no.	IC_50_ inhibitory concentration (nM/mL)^a^				
	ABTS^+^ radical^b^	Lipid peroxidation^c^	DPPH radical^d^	Superoxide anion^e^	Nitric oxide radical^f^
**9a**	73.30 ± 7.05* [4.07]	121.63 ± 18.60 [10.74]	134.07 ± 12.90* [22.34]	151.89 ± 14.42* [24.97]	103.67 ± 7.50* [12.99]
**9b**	93.30 ± 10.67* [6.16]	133.02 ± 11.53* [6.65]	88.19 ± 11.09* [6.40]	76.31 ± 11.80* [6.81]	52.57 ± 16.73* [9.66]
**9c**	196.17 ± 16.60* [9.58]	101.78 ± 14.51** [8.38]	41.27 ± 4.23** [2.44]	128.09 ± 21.74* [12.55]	81.90 ± 10.44* [6.02]
**9d**	55.61 ± 6.98* [4.03]	164.49 ± 14.56* [8.41]	63.56 ± 8.35** [4.82]	74.52 ± 8.3* [4.79]	53.03 ± 6.74* [3.89]
**9e**	47.89 ± 9.90* [5.72]	134.34 ± 14.70** [8.49]	107.28 ± 18.13** [10.46]	135.52 ± 22.55* [13.02]	155.21 ± 17.64* [10.19]
**9f**	207.14 ± 17.41* [10.05]	203.74 ± 20.11** [11.61]	80.63 ± 11.38** [6.57]	36.6604 ± 14.39* [8.31]	38.00 ± 11.77* [6.79]
Std	84.54 ± 9.39* [5.42]	150.12 ± 16.93** [9.77]	187.20 ± 35.38* [19.96]	171.36 ± 9.10** [5.25]	73.67 ± 9.44* [5.45]

* *P* < 0.05; ** *P* < 0.01

^a^IC_50_ value reported as Conc. ± SD [SEM]; SEM of three independent experiments performed in duplicate

^b^Standard used was trolox

^c^Standard used was ascorbic acid

^d^Standard used was ascorbic acid

^e^Standard used was catechin

^f^Standard used was curcumin

### Antimitotic activity

The levels of the physicochemical parameters of *Allium cepa* (root number and root length) were recorded after treatment with various drugs at 0, 48 and 72 h and found to cause significant inhibition in the growth of roots in comparison with negative control and positive control. From the observations, it has been revealed that average root length in (**9f**) treatment group was decreased significantly (1.06 cm) compared with that of the negative control (3.93 cm) after 72 h of treatment. The root morphology was nearly normal during the negative control treatment, but at positive control and synthesized compound groups, the roots morphology showed an obvious difference in its appearance in that it turned to slightly yellowish to brownish in colour. Its cytotoxic effect was evident in the form of shortening and decaying of roots, while progressive increases in root length and root numbers were observed in control group. The cytotoxic effect of tested compounds inhibits root growth and mitosis to a significant extent. The compound 9f showed lowest mitotic index (0.41 %) with highest activity among all the treatment groups, and it was also observed that the number of non-dividing cells increased in all treatment groups other than negative control. As there is no antimitotic principle in water, it was considered as negative control. Ethyl methanesulphonate (EMS) was treated as positive control treatment group and induces DNA damage by a direct mechanism, acting at various sites as a monofunctional ethylating agent of nucleotides (Budavari, 1989; Sega, 1984).

### Cytogenetic analysis

With the objective of investigating the possible mechanism involved in root growth inhibition, cytogenetic analysis was performed (Angayarkanni *et al*., 2007; Auti *et al*., 2010; Pavlica *et al*., 2000). All the tested compounds provoked strong inhibition of the mitotic index, where a statistically significant difference in relation to the control, and the decrease in the mitotic index was positively correlated with the electron-releasing group (Table 2). Changes in chromosome and cellular morphology were observed with increasing time. Partial c-mitosis (colchicine-like mitosis) and full c-mitosis, with partially functional spindles and completely normal mitotic phases, were seen in the various cells of the same root tip between 6- and 72-h time period. Cytogenetic alterations were investigated, and the results are depicted in Table 2. All the tested compounds induced chromosome and cytological alterations in treatment groups. An analysis of chromosome aberrations showed that most of the fragments detected in the different treatments were of chromosome type. The observation of chromosome breaks showed the clastogenic effect of tested compounds. The occurrence of chromosome fragments allows observation of statistically significant differences at tested synthesized compounds. In addition to the chromosome fragments, sticky metaphase and polar deviations (wrong directions of chromosome movement) were also observed. In general, it is possible to observe an increase in different abnormalities as the nucleophilic functional group concentration increased. In *Allium* test, a strong toxic effect of tested compounds was observed, supported by great occurrence of sticky metaphases, leading to cellular death (mitotic index decrease). All the tested compounds produced a significant decrease in mitotic index were time dependent at the treatment of 1 mg/mL. There was a statistically significant increase in total aberrant cells (*P* < 0.05) (aberrant cells include chromosome breaks, thickness and polar deviation) as compared with the negative control (Table 2); however, the highest value of aberrant cells is shown by the positive control. Statistical analysis showed that the genotoxic activities of the tested compounds induced micronuclei in the root tip meristem cells of *A. cepa.* Micronucleus formation in 1,000 cells per slide (‰MNC value) was also increased in tested compounds and in positive control EMS compared with negative control, which is statistically significant (*P* < 0.05).

**Table 2:** Mitotic index and chromosome and mitotic aberrations in the root meristem cells of *Allium cepa* after the synthesized compounds treatment

Treatment groups	Dose	MI (%) ± SEM^a,b^	Chromosome breaks (%) ± SEM^b^	Stickiness (%) ± SEM^b^	Polar deviations (%) ± SEM^b^	Aberrant cells (%) ± SEM^b^	MNC (‰) ± SEM^b^
NC^c^	–	6.22 ± 0.32	–	0.92 ± 0.32	6.89 ± 1.32	10.12 ± 1.58	0.35 ± 0.12
PC^d^	2 × 10^−2^ M	1.86 ± 0.23	–	36.31 ± 9.84	12.36 ± 3.36	43.20 ± 7.10	0.59 ± 0.09
**9a**	1 mg/mL	3.22 ± 0.16	6.22 ± 1.02	6.64 ± 2.38	8.62 ± 2.16	19.28 ± 5.22	0.34 ± 0.15
**9b**	1 mg/mL	2.77 ± 0.19	3.36 ± 0.57	9.12 ± 1.33	7.32 ± 1.24	24.64 ± 7.01	0.42 ± 0.18
**9c**	1 mg/mL	0.53 ± 0.03	–	28.04 ± 6.34	7.22 ± 2.61	38.54 ± 8.18	0.36 ± 0.14
**9d**	1 mg/mL	2.34 ± 0.19	0.96 ± 0.46	14.48 ± 2.52	9.15 ± 6.92	25.33 ± 9.42	0.51 ± 0.17
**9e**	1 mg/mL	1.27 ± 0.11	2.72 ± 0.94	9.88 ± 1.46	8.41 ± 1.35	26.74 ± 6.56	0.21 ± 0.06
**9f**	1 mg/mL	0.91 ± 0.13	1.47 ± 0.13	21.96 ± 7.22	7.33 ± 2.52	33.41 ± 9.47	0.39 ± 0.20
**9g**	1 mg/mL	0.41 ± 0.04	–	32.24 ± 6.92	10.26 ± 2.13	40.48 ± 12.94	0.48 ± 0.32
**9h**	1 mg/mL	1.07 ± 0.13	2.43 ± 0.67	16.50 ± 3.23	8.91 ± 1.56	29.83 ± 5.03	0.31 ± 0.14
**9i**	1 mg/mL	3.07 ± 0.22	7.33 ± 2.06	7.35 ± 2.06	6.57 ± 1.33	22.41 ± 6.18	0.61 ± 0.15

^a^The MI values indicates that lower the MI value higher the activity

^b^All the values are expressed as mean ± SEM “data are the mean, SEM of 3 independent experiments performed in duplicate”

^c^Distilled water was used as negative control (NC)

^d^Ethyl methanesulphonate (EMS) was used as positive control (PC)

In the light of the results obtained in the present study, these observations above may be due to the genotoxic and nucleotoxic action of the compounds or the disturbance of the formation of spindle fibres during cell division, which leads to chromosomal aberrations. Stickiness and clumping of the chromosomes were some of the most common effects of these tested compounds on the treated root tips. Stickiness usually leads to the formation of anaphase and telophase bridges, and this ends up inhibiting post-telophase, metaphase and cytokinesis, respectively, and thus hampering cell division.

#### In vitro cytotoxicity activity by MTT assay method

All the synthesized compounds prepared by Scheme I and previously reported (Chhajed *et al*., 2007, 2013) compounds were subjected to anticancer activity. CTC_50_ (cytotoxic concentration at which 50 % of the cells are dead after drug exposure) determined for test and standard compound with the help of MTT assay *HEK 293* (epidermal kidney cell line), *BT474* (breast cancer cell line) and *NCI*-*H226* (lung cancer) cell lines by MTT method (Freshney, 2000; Edmondson *et al*., 1988; Prasad *et al*., 2005; Chiruvella *et al*., 2008). The viability of control cells was designated as 100 %, and the others were expressed as percentage compared to the control. The results were compared with standard drug indisulam (ISL). The results demonstrated a strong dose-dependent growth inhibition in treated cell lines. It showed that different cells had a different sensitivity to the inhibition effect of tested compounds. The results are given in Tables 3, 4 and 5 *for HEK 293, BT474* and *NCI*-*H226*. Thus, from the data, it can be concluded that all test compounds are potent cytotoxic agents because of higher CTC_50_ at lower concentrations, and moreover, the compound (**4b**) (CTC_50_ = 0.922) and compound (**7f**) (CTC_50_ = 0.754) were found to be most potent agent among all the compounds tested against *HEK 293*. While compounds (**9c**) (CTC_50_ = 0.751) and (**9j**) (CTC_50_ = 0.913) were found to be most potent agent among all the compounds tested against *BT474* and *NCI*-*H226* cell lines, respectively. But none of tested compound was found to be potent compared to standard drug indisulam. From above all cell lines such as *HEK 293*, *M468* and *NCI*-*H226,* it has been concluded that compounds (**7f**), (**6f**), (**9b**), (**9c**) and (**9j**) are more potent than all synthesized compounds. Compounds (**6e**) and (**6b**) have moderate activity than all synthesized compounds. Compounds (**4a**) and (**9** **g**) have less activity than all synthesized compounds on all cell lines. Structure activity relationship of compounds showed that the presence of NH linker between aryl moiety which is substituted by electron-withdrawing group and 1,3,4-thiadiazole ring has been recognized as potent anticancer agent. Substitution on phenyl ring with chloro, methoxy and nitro group gives better anticancer activity.

**Table 3:** Anticancer activity (% cytotoxicity) and CTC_50_ values of synthesized compounds on HEK 293 (human epidermal kidney cell line)

Treatment	% cytotoxicity (100 − % cell survival) of HEK 293 cell line at conc. (μM)										CTCC_50_ (μM)^a^
	100	33.33	11.11	3.7	1.23	0.41	0.13	0.045	0.015	0.005	
	Log conc.										
	2.00	1.52	1.05	0.57	0.09	−0.39	−0.89	−1.35	−1.82	−2.30	
**4a**	32.96	31.71	29.48	28.87	28.54	28.18	26.93	26.64	25.82	25.57	64.363
**4b**	65.41	63.14	62.32	59.72	58.13	57.56	53.61	50.42	47.02	41.45	0.922
**4c**	49.12	47.84	46.53	42.12	40.66	39.93	39.10	38.24	37.87	36.34	4.563
**4d**	48.13	47.57	47.04	44.62	42.39	42.08	40.54	39.42	38.30	37.27	10.347
**4e**	40.20	40.04	39.12	38.89	37.12	35.43	34.75	34.13	31.57	30.58	1.8846
**4f**	31.97	31.19	30.74	30.04	29.17	28.85	28.43	28.12	26.39	24.28	120.951
**4g**	50.18	48.71	47.08	46.35	45.62	45.14	43.74	41.18	40.53	39.32	2.798
**6a**	35.42	35.16	34.98	33.56	32.17	30.14	29.88	28.19	26.78	26.51	97.475
**6b**	48.23	46.83	45.29	43.99	43.13	42.63	39.91	37.86	36.22	35.64	4.324
**6c**	38.78	38.22	37.79	36.59	35.72	34.75	33.58	32.94	32.05	30.46	187.19
**6d**	41.30	40.73	39.29	38.41	37.16	36.73	35.94	35.10	34.80	33.32	31.793
**6e**	54.97	51.16	49.87	49.15	47.06	45.27	43.36	42.66	41.98	39.12	3.937
**6f**	62.43	59.31	58.65	54.16	51.24	49.12	47.20	45.35	42.21	39.29	1.122
**6g**	31.97	28.73	26.15	24.22	20.81	20.09	18.32	18.01	16.52	15.14	6.658
**7a**	35.69	34.15	33.49	32.54	32.45	30.16	28.58	26.39	25.75	23.69	5.525
**7b**	51.86	50.68	48.17	47.80	46.53	45.26	43.99	40.45	39.24	37.78	2.268
**7c**	49.93	49.17	49.15	47.06	45.27	43.36	42.66	40.65	38.21	36.49	4.621
**7d**	29.58	29.03	27.25	26.57	25.26	24.12	22.18	20.28	19.87	18.85	31.443
**7e**	39.76	38.78	38.08	36.42	35.48	34.68	32.12	30.19	28.97	26.94	2.337
**7f**	43.78	41.25	40.59	39.53	38.74	37.52	36.99	36.04	35.11	33.19	0.754
**7g**	42.87	40.29	38.13	37.17	36.52	35.91	35.14	33.26	31.16	29.12	1.261
**9a**	50.59	46.23	45.62	44.17	43.11	42.42	40.73	39.83	38.24	37.35	24.642
**9b**	40.72	38.89	38.60	38.21	38.04	37.73	36.59	34.57	34.08	33.23	1.162
**9c**	52.34	47.41	45.94	44.29	43.13	42.92	42.06	40.33	38.16	36.83	2.413
**9d**	38.89	38.22	36.31	35.84	35.51	34.78	34.75	33.85	32.57	30.64	12.77
**9e**	39.61	37.65	34.24	31.41	30.29	29.81	28.32	26.59	26.66	25.27	16.044
**9f**	42.81	39.79	37.94	37.43	37.11	36.42	35.14	34.03	33.12	32.53	7.428
**9g**	38.61	34.14	33.55	32.77	32.09	31.15	30.32	28.54	27.57	25.40	22.12
**9h**	37.59	36.90	36.25	35.73	35.68	35.06	34.82	34.54	32.93	32.02	1.829
**9i**	43.48	39.51	38.84	37.19	37.03	36.69	36.32	35.12	34.46	33.04	41.71
**9j**	38.91	36.86	36.12	35.26	35.02	34.51	34.31	33.73	32.81	31.41	2.934
ISL	69.39	61.24	57.83	55.37	52.22	51.07	50.12	48.56	46.89	42.28	0.217

^a^CTC_50_ cytotoxicity concentration (μM) determined experimentally

**Table 4:** Anticancer activity (% cytotoxicity) and CTC_50_ values of synthesized compounds on BT474 (breast cancer cell line)

Treatment	% cytotoxicity (100 − % cell survival) of BT474 cell line at conc. (μM)										CTC_50_ (μM)^a^
	100	33.33	11.11	3.7	1.23	0.41	0.13	0.045	0.015	0.005	
	Log conc.										
	2.00	1.52	1.05	0.57	0.09	−0.39	−0.89	−1.35	−1.82	−2.30	
**4a**	28.73	28.11	27.45	26.28	25.92	25.37	24.64	23.12	22.64	20.06	61.336
**4b**	26.66	23.31	22.19	20.47	19.85	18.14	17.99	17.37	16.56	16.18	5.496
**4c**	41.35	40.32	39.37	38.82	37.56	36.26	35.55	34.19	32.11	30.65	8.743
**4d**	32.09	30.34	29.44	28.10	27.13	26.82	26.23	25.34	24.24	23.19	1.746
**4e**	40.37	38.91	37.21	36.96	35.73	33.14	32.29	31.76	31.02	30.89	2.798
**4f**	59.31	55.26	52.38	50.12	48.54	45.32	43.76	41.28	39.05	37.60	1.561
**4g**	38.22	37.84	36.21	35.19	34.87	34.15	33.18	32.07	31.45	30.59	2.346
**6a**	32.69	32.09	31.26	30.89	30.38	29.83	28.61	27.96	27.18	26.01	11.147
**6b**	31.97	30.32	29.34	28.72	28.14	27.13	26.25	25.78	25.06	24.32	3.656
**6c**	39.44	38.21	37.91	37.09	36.69	35.37	34.95	34.13	33.27	33.11	11.552
**6d**	33.85	33.29	32.92	32.11	31.02	30.56	29.44	28.93	27.72	26.34	127.620
**6e**	37.27	34.77	32.45	31.08	30.13	29.38	28.67	28.11	28.01	27.14	2.418
**6f**	50.81	45.31	42.19	40.62	37.19	35.84	33.41	32.15	30.07	29.13	1.007
**6g**	46.38	44.19	42.44	39.51	38.20	37.56	34.12	33.86	32.75	30.46	1.028
**7a**	46.32	43.67	41.82	40.72	39.54	38.21	37.77	36.69	34.95	34.13	9.215
**7b**	36.61	35.52	34.59	33.33	32.16	31.36	30.24	29.47	28.13	27.42	1.884
**7c**	27.87	26.43	25.71	24.22	22.81	20.98	20.13	19.76	19.43	18.80	10.336
**7d**	38.89	37.95	36.07	35.68	34.42	33.11	31.92	30.64	29.31	28.53	1.195
**7e**	51.16	50.38	49.11	48.46	47.56	47.13	46.28	45.39	44.21	43.90	2.349
**7f**	64.14	60.28	58.64	56.72	54.23	52.17	50.09	47.21	45.80	42.38	0.751
**7g**	40.06	38.46	37.71	34.74	33.24	32.73	31.29	29.98	28.39	27.27	1.473
**9a**	65.97	41.46	40.56	40.2	38.97	38.05	37.05	36.38	35.84	35.26	13.723
**9b**	64.99	62.26	60.68	56.34	50.12	46.10	42.01	41.47	39.42	38.81	2.414
**9c**	67.11	58.80	54.83	53.61	50.42	47.02	44.37	42.60	41.45	38.13	0.794
**9d**	39.40	38.00	37.37	36.80	36.75	34.22	33.96	33.52	33.42	33.28	11.557
**9e**	56.21	47.52	41.77	37.86	31.92	29.89	28.93	27.27	26.43	25.17	12.770
**9f**	38.66	38.22	36.12	35.80	35.51	34.78	34.75	33.86	32.57	30.64	112.202
**9g**	38.14	36.17	34.74	33.23	32.82	31.42	29.23	28.71	28.02	27.38	18.345
**9h**	47.67	41.55	38.42	35.17	34.21	33.76	32.92	30.64	29.11	29.02	1.281
**9i**	41.29	40.50	39.19	37.56	36.73	36.12	35.42	34.59	33.31	31.52	6.324
**9j**	61.43	56.93	52.13	49.34	45.14	43.57	40.13	37.35	34.64	30.38	1.361
ISL	73.52	66.14	62.46	54.71	52.94	50.79	49.03	46.42	44.97	42.23	0.348

^a^CTC_50_ cytotoxicity concentration (μM) determined experimentally

**Table 5:** Anticancer activity (% cytotoxicity) and CTC_50_ values of synthesized compounds on NCI-H226 (lung cancer cell line)

Treatment	% Cytotoxicity (100 − % cell survival) of NCI-H226 cell line at conc. (μM)										CTC_50_ (μM)^a^
	100	33.33	11.11	3.7	1.23	0.41	0.13	0.045	0.015	0.005	
	Log conc.										
	2.00	1.52	1.05	0.57	0.09	−0.39	−0.89	−1.35	−1.82	−2.30	
**4a**	27.86	27.49	27.09	26.55	26.13	25.46	24.94	24.22	23.14	22.41	145.347
**4b**	39.46	37.21	36.30	35.96	35.11	34.69	34.05	33.46	32.87	32.12	33.268
**4c**	54.16	51.24	49.12	47.20	45.35	42.21	39.29	38.91	38.19	37.65	2.527
**4d**	42.56	42.06	39.73	38.26	38.02	37.34	36.29	35.23	35.11	34.16	17.482
**4e**	34.59	33.33	32.16	31.26	30.59	29.89	29.55	28.93	28.11	27.31	7.965
**4f**	52.87	50.14	48.31	46.52	45.78	42.90	41.57	39.72	38.63	37.24	1.197
**4g**	41.75	41.35	40.29	39.37	38.14	37.84	37.13	36.95	36.10	35.77	22.274
**6a**	40.92	38.89	38.22	36.05	35.61	33.65	32.94	32.17	31.57	30.46	52.953
**6b**	49.15	47.06	45.27	43.36	42.66	41.98	39.12	38.44	37.26	36.29	1.119
**6c**	38.98	38.32	36.52	35.08	34.91	34.79	34.15	33.59	32.75	30.41	12.829
**6d**	49.15	47.26	45.31	43.41	41.96	41.18	39.12	37.05	36.38	35.51	1.816
**6e**	54.52	51.14	50.83	49.22	48.64	47.65	45.39	42.38	41.25	38.76	1.018
**6f**	65.97	59.62	57.09	55.18	54.64	51.26	48.28	46.54	44.85	41.28	0.978
**6g**	46.01	43.19	42.63	41.32	40.65	39.82	37.34	36.75	34.95	33.52	3.108
**7a**	36.94	36.21	35.13	34.55	32.17	30.41	29.35	29.17	28.36	27.44	10.735
**7b**	42.44	41.12	40.65	39.07	38.79	37.41	37.05	35.48	33.62	33.48	13.829
**7c**	40.27	38.88	38.60	38.21	38.04	37.79	36.59	34.75	34.03	33.23	1.164
**7d**	38.92	38.50	37.91	35.98	35.37	35.66	35.17	34.59	34.13	33.72	6.342
**7e**	36.05	35.80	35.53	34.87	34.52	33.48	31.75	30.46	29.97	29.04	12.729
**7f**	67.99	65.83	60.68	56.43	52.12	46.10	42.62	40.07	39.26	38.76	1.784
**7g**	38.99	38.74	37.12	36.26	36.11	35.72	35.32	33.62	32.79	30.66	10.215
**9a**	42.36	41.13	39.07	38.10	37.89	37.01	36.15	35.32	34.84	33.29	5.674
**9b**	37.99	37.72	37.02	36.62	36.47	36.11	35.72	35.43	29.46	27.75	1.487
**9c**	43.51	40.34	38.19	37.73	36.15	35.87	35.12	34.15	33.25	31.49	5.726
**9d**	53.02	48.22	47.78	43.14	41.21	40.59	38.31	37.46	36.27	35.65	2.268
**9e**	51.36	49.32	48.22	47.61	45.79	43.35	42.54	41.86	40.27	39.11	12.763
**9f**	40.39	38.72	37.14	36.91	35.67	34.95	33.42	32.39	31.24	30.26	17.327
**9g**	42.47	39.75	39.20	38.61	37.51	36.33	35.06	34.11	33.17	32.72	166.376
**9h**	39.98	39.25	37.94	37.46	37.24	36.39	36.32	35.35	35.01	32.85	1.467
**9i**	38.66	38.57	36.72	35.27	34.95	34.59	34.14	33.97	33.92	33.61	9.215
**9j**	52.43	45.35	42.72	39.13	37.04	36.06	35.27	34.62	33.23	32.98	0.913
ISL	59.26	44.69	38.58	36.46	34.12	32.98	31.11	30.20	28.42	26.37	0.313

^a^CTC_50_ cytotoxicity concentration (μM) determined experimentally

The order of cytotoxic activity was electron-withdrawing group on phenyl > electron-donating group on phenyl > phenyl.

We can conclude that electron-releasing group on phenyl ring is responsible for less activity.

## Conclusion

Thiadiazoles are mesoionic system, a poly-heteroatomic system containing a five-membered heterocycle associated with a conjugation of p and π electrons and distinct regions of positive and negative charges leading to highly polarizable derivatives. This distinctive characteristic allows mesoionic compounds to effectively cross-cellular membranes and interact with biological molecules in unique ways. The good liposolubility of the sulphur atom in the heterocycle might also have a positive effect on the biological activity and pharmacokinetic properties of thiadiazole-containing compounds. The thiadiazole ring possesses similar chemical properties to the pyrimidine ring and can be considered a bioisostere. Given that the pyrimidine structure is found in nucleobases, components of nucleotides and the building blocks of DNA and RNA, it seems likely that thiadiazole could readily interact with DNA and RNA, potentially explaining the broad and often potent activity. Furthermore, this activity against DNA suggests that thiadiazoles derivatives could potentially be used for chemical intervention at the gene level. Compounds containing thiadiazole with high potency have been reported here, and some of them displayed excellent activities against a range of tumour cells. The ability of thiadiazoles to target DNA could explain their potential anticancer activity as uncontrolled DNA replication/cell division is a hallmark of neoplastic diseases. Furthermore, the heteroatoms of the thiadiazole are able to form interactions, such as hydrogen bonds, with biological targets that include key kinases that participate in tumorigenesis, such as CA IX and XII.

The sulfonyl group of sulphonamides is similar to the carbonate ion and can competitively inhibit CAs. Compounds containing a thiadiazole, a benzene bioisostere, should also possess high inhibitory activity when bonded with a sulphamide group. From lead compound, acetazolamide, some of the most potent compounds were synthesized and evaluated several sulphonamides as inhibitors of in vitro cancer cell growth compared with selective hCA IX inhibitor, indisulam. The affinity of 1,3,4-thiadiazole for hCA increases significantly when substituted with sulphonamides connected with Schiff base. These results indicate that the thiadiazole ring has receptor-binding ability in the context of hCA IX inhibition and in the prevention of cancer associated with CA.

## Experimental section

### Synthetic study

Melting points were determined in one-end-open capillary tubes on a Thermonik Precision melting point apparatus (C-PMP-2, Mumbai, India) and presented without any corrections. The IR spectra ($\tilde{\nu}$ , cm^−1^) were recorded in KBr tablets using Shimadzu FT-IR 8400s spectrophotometer. ^1^H nuclear magnetic resonance (^1^H-NMR) spectra were recorded for the compounds on Varian EM-390 apparatus by using TMS as an internal standard. ^13^C-NMR spectra were recorded for the compounds on Bruker Avance II 400 NMR Spectrometer apparatus using TMS as an internal standard, and chemical shifts are reported in ppm (*δ*-scale).

Elemental analysis of the obtained compounds was performed for C, H, N, S using Elemental Vario EL III Carlo Erba 1106 analyzer. The maximum percentage differences between calculated and found values for each element were within the error and amounted to ±0.4 %. The completion of reaction and the purity of the obtained compounds were checked by TLC on aluminium oxide 60 F254 plates (Merck Co., Whitehouse Station, NJ, USA), in a CHCl3/C2H5OH (3:1, v/v) solvent system. The spots were developed in iodine chamber and visualized under ultra violet lamp (*λ* = 254 nm).

### General procedure

*N*-[(5-Amino-1,3,4-thiadiazol-2-yl)sulfonyl]benzamide (**4**); 5-[(4-acetamido)benzene sulphonamido]-1,3,4-thiadiazol-2-(*N*-benzoyl)sulphonamide (**6**); and 5-[(4-amino)benzene sulphonamido]-1,3,4-thiadiazol-2-(*N*-benzoyl)sulphonamide (**7**) were synthesized, and their physicochemical and spectral data were reported previously (Chhajed *et al*., 2007, 2013). The synthesis outline is depicted in Scheme 1.

#### *N*-{[5-(Benzylidenamino)-1,3,4-thiadiazol-2-yl]sulphonyl} benzamide (**9a**)

Offwhitecrystals (EtOH) (this compound was prepared by refuxing 5-amino-1,3,4-thiadiazol-2-[*N*-(benzoyl)]sulphonamide (2.74 g, 0.01 mol) (**4a**) and benzaldehyde (**8a**) (1.06 g, 0.01 mol) in ethanol (20 mL) using 2–3 drops of sulphuric acid as catalyst, for 12 h. Pour it with thin stream into crushed ice. It was obtained as yellowish coloured solid and recrystallized by ethanol); yield: 63 %; Mp: 185–187 °C; UV (MeOH) *λ*_max_ (log *ε*) 287 nm; *R*_*f*_ = 0.62 (CHCl_3_/EtOH, 3/1); FT-IR (KBr): *v*_max_ 3,625.1, 3,037.4, 1,693.4, 1,678.7, 1,624.32, 1,598.4, 1,557.7, 1,517–1,530.9, 1,369.6, 1,290.5, 907.25, 764.44, 756.54, 694.91 cm^−1^; ^1^H-NMR (DMSO, 400 MHz): *δ* = 1.257 (1H, s, –CH–), 2.134 (6H, m, CH–C_6_H_5_), 2.590 (6H, m, CO–C_6_H_5_), 3.965 (1H, s, CH=N), 4.18 (1H, s, N–H), 7.664–7.685 ppm (10H, m, Ar–H); ^13^C-NMR ([D]_6_DMSO, 75 MHz): *δ* = 171.46 (C, amide), 168.56 (C_2_, thiadiazole), 166.67 (C_5_, thiadiazole), 160.68 (C, imine), 137.78 (C_1_, Ar′–C-imine), 136.05 (C_1_, Ar–C-amide), 134.24 (C_4_, CH–Ar′), 132.52 (C_3_, CH–Ar), 131.71 (C_3_, CH–Ar′), 130.39 (C_5_, CH–Ar), 129.29 (C_2_, CH–Ar′), 129.15 (C_6_, CH–Ar′), 128.84 (C_2_, CH–Ar), 128.42 (C_6_, CH–Ar), 127.34 (C_5_, CH–Ar′); EIMS m/z [M]^+^ 370.9 (100); Anal. Calcd. for C_16_H_12_N_4_O_3_S_2_: C, 51.60; H, 3.25; N, 15.04; S, 17.22. Found: C, 51.61; H, 3.24; N, 15.05; S, 17.22.

#### *N*-({5-[(4-Chlorobenzylidene)amino]-1,3,4-thiadiazol-2-yl}sulfonyl)benzamide (**9b**)

Yield: 64.2 %: Mp: 212–214 °C; *λ*_max_ (log *ε*) 305 nm; *R*_*f*_ = 0.65 (CHCl_3_/EtOH, 3/1); FT-IR (KBr): *v*_max_ 3,465.3, 3,417.47, 3,148.51, 1,673.2–1,668.7, 1,624.32–1,598.4, 1,545.9, 1,538.1–1,527.4, 1,368.9–1,358.8, 1,169.9, 968.07, 848–826.5, 764.43–674.43, 764.43 cm^−1^; ^1^H-NMR (DMSO, 400 MHz): *δ* = 1.359 (1H, s, –CH–), 2.342 (6H, m, CH–C_6_H_5_), 2.678 (6H, m, CO–C_6_H_5_), 3.623 (1H, s, CH=N), 4.41 (1H, s, N–H), 7.462–8.104 (10H, m, Ar–H) 8.24- 8.362 ppm (1H, s, C(=O)N–H); ^13^C–NMR ([D]_6_DMSO, 75 MHz): *δ* = 170.64 (C, amide), 168.41 (C_5_, thiadiazole), 166.58 (C_2_, thiadiazole), 161.68 (C, imine), 136.24 (C_4_, Cl–C–Ar′), 134.16 (C_1_, Ar–C-amide), 133.78(C_1_, Ar′–C-imine), 130.25 (C_4_, CH–Ar), 129.15 (C_3_, CH–Ar′), 129.29 (C_5_, CH–Ar′), 129.02 (C_3_, CH–Ar), 128.97 (C_5_, CH–Ar), 128.84 (C_2_, CH–Ar′), 128.42 (C_6_, CH–Ar′), 127.34 (C_2_, CH–Ar), 127.29 (C_6_, CH–Ar); EIMS m/z [M]^+^ 412.9 (100); Anal. calcd. for C_16_H_11_N_4_O_3_S_2_Cl: C, 47.23; H, 2.73; N, 13.77; S, 15.76. Found: C, 47.24; H, 2.72; N, 13.75; S, 15.77.

#### *N*-({5-[(2-Methoxybenzylidene)amino]-1,3,4-thiadiazol-2-yl}sulfonyl)benzamide (**9c**)

Yield: 62.8 %; Mp: 201–203 °C; UV (MeOH) *λ*_max_ (log *ε*) 315 nm; *R*_*f*_ = 0.57 (CHCl_3_/EtOH, 3/1); FT-IR (KBr): *v*_max_ 3,625.4, 3,048.7, 2,915.3–2,903.2, 1,692.8, 1,681.1–1,665.4, 1,599.9–1,536.5, 1,426.5, 1,347.1, 1,290, 1,143.2–1,129.4, 930.13–923.7, 762.6–713.1, 762.6 cm^−1^ (thiadiazole C–N stretching); ^1^H-NMR (DMSO, 400 MHz): *δ* = 1.352 (1H, s, –CH–), 3.134 (1H, s, CH–C_6_H_5_), 3.417–3.487 (3H, m, –OCH_3_), 6.364 (1H, s, Ar′–H_3,5_), 6.84–7.16 (3H, *J* = 7.2 Hz, t, Ar–H_3,4,5_), 8.285 (2H, *J* = 2.4 Hz, d, Ar–H_2,6_), 8.58 ppm (1H, s, N–H); ^13^C-NMR ([D]_6_DMSO, 75 MHz): *δ* = 168.21(C, amide), 164.03 (C_2_, C–Ar′–OCH_3_), 163.77(C, imine), 162.32 (C_2_, thiadiazole), 162.28 (C_5_, thiadiazole), 134.25(C_1_, CH–Ar), 132.22 (C_4_, CH–Ar), 130.76 (C_4_, CH–Ar′), 130.32 (C_6_, CH–Ar′), 128.66 (C_3_, CH–Ar), 128.45 (C_5_, CH–Ar), 128.23 (C_1_, CH–Ar′), 127.55 (C_2_, CH–Ar), 127.46 (C_6_, CH–Ar), 120.84 (C_3_, CH–Ar′), 120.44 (C_5_, CH–Ar′), 62.32 (C, aliphatic, OCH3) ppm; EIMS m/z [M]^+^ 404.6 (100); Anal. calcd. for C_17_H_14_N_4_O_4_S_2_: C, 50.74; H, 3.51; N, 13.92; S, 15.93. Found: C, 50.74; H, 3.52; N, 13.95; S, 15.92.

#### *N*-({5-[(4-Methoxybenzylidene)amino]-1,3,4-thiadiazol-2-yl}sulfonyl)benzamide (**9d**)

Yield: 65.3 %; Mp: 215–217 °C; *λ*_max_ (log *ε*) 287 nm; *R*_*f*_ = 0.45 (CHCl_3_/EtOH, 3/1); FT-IR (KBr): *v*_max_ 3,659.8–3,625.4, 2,915.3–2,903.2, 2,884.5, 1,692.8, 1,681.1–1,665.4, 1,599.9–1,536.5, 1,426.5, 1,347.1, 1,290–1,274.4, 1,143.2–1,013.4, 930.13–923.7, 786.79–762.6, 762.6 cm^−1^; ^1^H-NMR (DMSO, 400 MHz): *δ* = 3.721 (3H, s, –OCH_3_), 6.463 (2H, s, Ar′–H_3,5_), 7.331–7.62 (5H, *J* = 3.0 Hz, d, Ar–H), 8.125 (3H, s, Ar–H_2,6_), 8.24 ppm (1H, s, C(=O)N–H); ^13^C-NMR ([D]_6_DMSO, 75 MHz): *δ* = 170.34 (C, amide), 165.29 (C_4_, C–Ar′-OCH_3_), 163.51 (C, imine), 162.85 (C_2_, thiadiazole), 162.34 (C_5_, thiadiazole), 134.29(C_1_, CH–Ar), 134.01 (C_4_, CH–Ar), 130.49 (C_6_, CH–Ar′), 130.11 (C_2_, CH–Ar′), 128.94 (C_3_, CH–Ar), 128.22 (C_5_, CH–Ar), 128.11 (C_1_, CH–Ar′), 127.42 (C_2_, CH–Ar), 127.16 (C_6_, CH–Ar), 114.33 (C_5_, CH–Ar′), 114.08 (C_3_, CH–Ar′), 69.41 (C, OCH3) ppm; EIMS m/z [M]^+^ 403.9 (100); Anal. calcd. for C_17_H_14_N_4_O_4_S_2_: C, 50.74; H, 3.51; N, 13.92; S, 15.93. Found: C, 50.72; H, 3.52; N, 13.96; S, 15.94.

#### *N*-({5-[(4-Hydroxybenzylidene)amino]-1,3,4-thiadiazol-2-yl}sulfonyl)benzamide (**9e**)

Yield: 68.2 %; Mp: 178–180 °C; UV (MeOH) *λ*_max_ (log *ε*) 375 nm; *R*_*f*_ = 0.59 (CHCl_3_/EtOH, 3/1); FT-IR (KBr): *v*_max_ 3,769–3,719.8, 3,671.56–3,523.8, 2,884.5, 1,713.8, 1,673.7–1,665.4, 1,599.9–1,549, 1,454.6–1,424.2, 1,317.8, 1,292–1,174.8, 1,174.8–1,052.1, 931.21–921.7, 786.79–762.6, 761.6–725.58 cm^−1^; ^1^H-NMR (400 MHz, DMSO): *δ* = 3.569 (1H, s, CH=N), 4.684 (1H, s, –OH), 6.547–8. 623 (9H, m, Ar–H), 8.31 ppm (1H, s, C(=O)N–H); ^13^C-NMR ([D]_6_DMSO, 75 MHz): *δ* = 169.43 (C, imine), 167.11(C, amide), 161.32 (C_4_, C–Ar′–OH), 161.02 (C_2_, thiadiazole), 160.98 (C_5_, thiadiazole), 134.52 (C_1_, CH–Ar), 131.17 (C_4_, CH–Ar), 130.62 (C_6_, CH–Ar′), 130.26 (C_2_, CH–Ar′), 128.82 (C_3_, CH–Ar), 128.29 (C_5_, CH–Ar), 127.34 (C_1_, CH–Ar′), 127.55 (C_2_, CH–Ar), 127.21 (C_6_, CH–Ar), 114.83 (C_5_, CH–Ar′), 114.12 (C_3_, CH–Ar′), ppm; EIMS m/z [M]^+^ 386.6 (100); Anal. calcd. for C_16_H_12_N_4_O_4_S_2_: C, 49.48; H, 3.11; N, 14.42; S, 16.51. Found: C, 49.50; H, 3.12; N, 14.40; S, 16.51.

#### *N*-({5-[(2-Hydroxybenzylidene)amino]-1,3,4-thiadiazol-2-yl}sulfonyl)benzamide (**9f**)

Yield: 64.6 %; Mp: 220–222 °C; UV (MeOH) *λ*_max_ (log *ε*) 478 nm; *R*_*f*_ = 0.64 (CHCl_3_/EtOH, 3/1); FT-IR (KBr): *v*_max_ 3,489.1, 3,261.43, 2,948.5–2,884.5, 1,731.22–1,635.4, 1,614.217–1,589, 1,436.06–1,505.64, 1,330.70, 1,232.41–1,093.86, 1,093.86, 974.20–841.7, 822.2–780.44, 761.6–725.58 cm^−1^; ^1^H-NMR (400 MHz, DMSO): *δ* = 3.582 (1H, s, CH = N), 4.237 (1H, s, –OH), 6.413–8.548 (9H, m, Ar–H), 8.41 ppm (1H, s, C(=O)N–H); ^13^C-NMR ([D]_6_DMSO, 75 MHz): *δ* = 166.14 (C, imine), 165.26 (C, amide), 164.21 (C, C_2_–Ar′–OH), 160.72 (C_5_, thiadiazole), 160.19 (C_2_, thiadiazole), 134.82 (C_1_, CH–Ar), 132.77 (C_4_, CH–Ar′), 131.38 (C_4_, CH–Ar), 130.15 (C_6_, CH–Ar′), 128.81 (C_3_, CH–Ar), 128.49 (C_5_, CH–Ar), 128.09 (C_5_, CH–Ar′), 127.40 (C_2_, CH–Ar), 127.12 (C_6_, CH–Ar), 114.52 (C_1_, CH–Ar′), 114.33 (C_3_, CH–Ar′), ppm; EIMS m/z [M]^+^ 389.4 (100); Anal. calcd. for C_16_H_12_N_4_O_4_S_2_: C, 49.48; H, 3.11; N, 14.42; S, 16.51. Found: C, 49.47; H, 3.12; N, 14.43; S, 16.52.

#### *N*-({5-[(4-Hydroxy-3-methoxy benzylidene)amino]-1,3,4-thiadiazol-2-yl}sulfonyl)benzamide (**9g**)

Yield: 64.2 %; Mp: 252–254 °C; UV (MeOH) *λ*_max_ (log *ε*) 268 nm; *R*_*f*_ = 0.67 (CHCl_3_/EtOH, 3/1); FT-IR (KBr): *v*_max_ 3,537.42, 3,371.43, 2,927.5–2,853.4, 1,692.8–1,681.1, 1,665.4–1,599.9, 1,536.05–1,426.5, 1,347.1–1,290, 1,274.4–1,182.6, 1,013.4, 930.13–923.7, 844.17–762.6, 762.6–713.1 cm^−1^; ^1^H-NMR (400 MHz, DMSO): *δ* = 3.069 (3H, s, –OCH_3_), 3.659 (1H, s, CH=N), 4.428 (1H, s, –OH), 6.126–8.262 (8H, m, Ar–H), 8.523 ppm (1H, s, C(=O)N–H); ^13^C-NMR ([D]_6_DMSO, 75 MHz): *δ* = 170.43 (C, imine), 167.67(C, amide), 165.09 (C_5_, thiadiazole), 164.18 (C_2_, thiadiazole), 154.32 (C_3_, C–Ar′–OCH3), 145.13 (C_4_, C–Ar′–OH), 135.14 (C_1_, CH–Ar), 134.02 (C_4_, CH–Ar), 128.83 (C_3_, CH–Ar), 128.41 (C_5_, CH–Ar), 127.34 (C_1_, CH–Ar′), 127.21 (C_2_, CH–Ar), 121.62 (C_6_, CH–Ar′), 117.61 (C_6_, CH–Ar), 117.26 (C_5_, CH–Ar′), 114.31 (C_2_, CH–Ar′), 65.17 (C, Ar–OCH_3_), ppm; EIMS m/z [M]^+^ 420.1 (100); Anal. calcd. for C_17_H_14_N_4_O_5_S_2_: C, 48.80; H, 3.37; N, 13.39; S, 15.33. Found: C, 48.78; H, 3.38; N, 13.41; S, 15.34.

#### *N*-[(5-{[4-(Dimethylamino)benzylidene]amino}-1,3,4-thiadiazol-2-yl)sulfonyl]benzamide (**9h**)

Yield: 67.7 %; Mp: 236–238 °C; UV (MeOH) *λ*_max_ (log *ε*) 305 nm; *R*_*f*_ = 0.42 (CHCl_3_/EtOH, 3/1); FT-IR (KBr): *v*_max_ 3,652.4, 3,532.12, 3,114.7, 2,985.3–2,896.4, 1,614.2–1,591.4, 1,413.1, 1,238.52–1,174.7, 804.2–783.6, 743.9–719.2 cm^−1^; ^1^H-NMR (400 MHz, DMSO): *δ* = 2.547 (6H, s, –NCH_3_), 3.956 (1H, s, CH=N), 4.114 (1H, s, N–H), 6.466–7.824 (9H, m, Ar–H), 8.511 ppm (1H, s, C(=O)N–H); ^13^C-NMR ([D]_6_DMSO, 75 MHz): *δ* = 169.42 (C, imine), 165.21 (C, amide), 162.15 (C_2_, thiadiazole), 162.11 (C_5_, thiadiazole), 154.32 (C_4_, C–Ar′–N(CH_3_)_2_), 134.63 (C_1_, CH–Ar), 132.46 (C_4_, CH–Ar), 132.23 (C_2_, CH–Ar′), 132.18 (C_3_, CH–Ar), 131.65 (C_6_, CH–Ar′), 128.12 (C_2_, CH–Ar), 128.03 (C_6_, CH–Ar), 127.37 (C_1_, CH–Ar′), 127.11 (C_3_, CH–Ar′), 117.52 (C_5_, CH–Ar), 117.11 (C_5_, CH–Ar′), 52.84 (C, Ar–NCH_3_, Aliphatic), 52.47 (C, Ar–NCH_3_, Aliphatic) ppm; EIMS m/z [M]^+^ 415.7 (100); Anal. calcd. for C_19_H_18_N_4_O_3_S_2_: C, 55.06; H, 4.38; N, 13.52; S, 15.47. Found: C, 55.07; H, 4.38; N, 13.53; S, 15.46.

#### *N*-({5-[(3-Nitrobenzylidene)amino]-1,3,4-thiadiazol-2-yl}sulfonyl)benzamide (**9i**)

Yield: 61.3 %; Mp: 258–260 °C; UV (MeOH) *λ*_max_ (log *ε*) 352 nm; *R*_*f*_ = 0.51 (CHCl_3_/EtOH, 3/1); FT-IR (KBr): *v*_max_ 3,537.9–3,427.2, 3,128.2–3,022.3, 3,075–3,007.4, 2,341.6–2,331.1, 1,445.8, 1,456.8–1,531.7, 827, 1,022.8–1,078.2, 713.1–619.5 cm^−1^; ^1^H-NMR (400 MHz, DMSO): *δ* = 3.239 (1H, s, CH=N), 4.751 (1H, s, –OH), 6.872–8.421 (9H, m, Ar–H), 8.645 ppm (1H, s, C(=O)N–H); ^13^C-NMR ([D]_6_DMSO, 75 MHz): *δ* = 168.27 (C, imine), 165.61 (C, amide), 162.23 (C_5_, thiadiazole), 162.18 (C_2_, thiadiazole), 154.32 (C_3_, C–Ar′–NO_2_), 135.71 (C_6_, CH–Ar′), 134.67 (C_1_, CH–Ar′), 134.46 (C_1_, CH–Ar), 132.49 (C_4_, CH–Ar), 129.37 (C_5_, CH–Ar′), 128.35 (C_3_, CH–Ar), 128.22 (C_5_, CH–Ar), 126.13 (C_4_, CH–Ar′), 117.11 (C_2_, CH–Ar′), 116.37 (C_2_, CH–Ar), 116.16 (C_6_, CH–Ar) ppm; EIMS m/z [M]^+^ 416.9 (100); Anal. calcd. for C_16_H_11_N_5_O_5_S_2_: C, 46.04; H, 2.66; N, 16.78; S, 15.36. Found: C, 46.05; H, 2.68; N, 16.80; S, 15.36.

#### *N*-({5-[(Furan-2-ylmethylidene)amino]-1,3,4-thiadiazol-2-yl}sulfonyl)benzamide (**9j**)

Brownish crystals (EtOH) (this compound was prepared by refuxing 5-amino-1,3,4-thiadiazol-2-[*N*-(benzoyl)]sulphonamide (2.74 g, 0.01 mol) (**4a**) and Furfuldehyde (**8j**) (0.96 g, 0.01 mol) in ethanol (20 mL) using 2–3 drops of sulphuric acid as catalyst, for 7 h. Pour it with thin stream into crushed ice. It was obtained as dark brown coloured solid and recrystallized by ethanol); Yield: 53.04 %; Mp: 261–263 °C; UV (MeOH) *λ*_max_ (log *ε*) 412 nm; *R*_*f*_ = 0.69 (CHCl_3_/EtOH, 3/1); FT-IR (KBr): *v*_max_ 3,634.9, 3,581.22, 3,054.2, 1,635.34, 1,622.4–1,595.9, 1,432.4, 1,254.31–1,197.7, 824.3–776.9, 741.3–711.4 cm^−1^; ^1^H-NMR (400 MHz, DMSO): *δ* = 2.547 (6H, s, –NCH_3_), 4.116 (1H, s, CH=N), 6.724–7.211 (3H, m, furfuryl-H), 7.446–7.918 (5H, m, Ar–H), 8.426 ppm (1H, s, C(=O)N–H); ^13^C-NMR ([D]_6_DMSO, 75 MHz): *δ* = 148.22 (C, imine), 167.19 (C, amide), 154.32 (C_2_, C-furfuryl), 152.13 (C_2_, thiadiazole), 150.84 (C_5_, thiadiazole), 135.71 (C_5_, CH-furfuryl), 134.63 (C_1_, CH–Ar), 132.46 (C_4_, CH–Ar), 128.12 (C_3_, CH–Ar), 128.03 (C_5_, CH–Ar), 117.11 (C_3_, CH-furfuryl), 111.24 (C_2_, CH–Ar), 111.06 (C_6_, CH–Ar), 106.10 (C_4_, CH-furfuryl) ppm; EIMS m/z [M]^+^ 364.3 (100); Anal. calcd. for C_14_H_10_N_4_O_4_S_2_: C, 46.40; H, 2.78; N, 15.46; S, 17.70. Found: C, 46.42; H, 2.79; N, 15.45; S, 17.39.

### Pharmacological evaluation

#### Antioxidant and free radical scavenging activity

##### Total antioxidant activity

The ability of the test sample to scavenge 2,2′-azinobis-(3-ethylbenzothiazoline-6-sulphonic acid) (ABTS^**·**+^) radical cation was compared with 6-hydroxy-2,5,7,8-tetramethylchroman-2-carboxylic acid (trolox) standard (Chang *et al*., 2007; Erel, 2004; Re *et al*., 1999). The ABTS^**·**+^ radical cation was pregenerated by mixing ABTS stock solution (7 mM) with potassium persulphate (2.45 mM) (final concentration) and incubating for 12–16 h in the dark at room temperature until the reaction was complete and the absorbance was stable. The absorbance of the ABTS^·+^ solution was equilibrated to 0.70 (±0.02) by diluting with water at room temperature, then 1 mL of solution was mixed with 10 μL of the test sample (0.05–10 mg/mL), and the absorbance was measured at 734 nm after 6 min. All experiments were repeated three times. The percentage inhibition of absorbance was calculated and plotted as a function of the concentration of standard and sample to determine the trolox equivalent antioxidant concentration (TEAC). To calculate the TEAC, the gradient of the plot for the sample was divided by the gradient of the plot for trolox. The IC_50_ inhibitory concentration (nM/mL) values of tested compounds are depicted in Table 1. The ABTS^**·**+^ radical scavenging activity of the samples was expressed as

$$S\,\% = [(A_{\text{control}} {-}A_{\text{sample}} )/A_{\text{control}} ] \times 100$$

where *A*_control_ is the absorbance of the blank control (ABTS^·+^ solution without test sample), and *A*_sample_ is the absorbance of the test sample.

##### Lipid peroxidation inhibitory activity

Egg lecithin (3 mg/mL phosphate buffer, pH 7.4) was sonicated in an ultrasonic sonicator for 10 min to ensure proper liposome formation. Test samples or standard, ascorbic acid (100 μL) of different concentrations (10, 20, 30, 40 50 and 100 μg/mL) was added to liposome mixture (1 mL); the control was without test sample. Lipid peroxidation was induced by adding ferric chloride (10 μL, 400 mM) and L-ascorbic acid (10 μL, 200 mM). After incubation for 1 h at 37 °C, the reaction was stopped by adding hydrochloric acid (2 mL, 0.25 N) containing trichloroacetic acid (150 mg/mL), thiobarbituric acid (3.75 mg/mL) and butylated hydroxy anisole (0.50 mg/mL). The reaction mixture was subsequently boiled for 15 min, cooled and centrifuged at 1,000 rpm for 15 min, and the absorbance of the supernatant was measured at 532 nm (Duh and Yen, 1997). The IC_50_ values of all tested compounds are reported in Table 1. The % inhibition at different concentrations was calculated by the following formula

$$\% \,{\text{Inhibition}} = [1 - (V_{\text{t}} /V_{\text{c}} )] \times 100$$

where *V*_t_ = mean absorption of test compound, *V*_c_ = mean absorption of control.

The IC_50_ (nM/mL) value was derived from the % inhibition at different concentrations.

##### DPPH radical scavenging activity

Compounds of SC series were evaluated for their in vitro free radical scavenging activities by 2,2-diphenyl-1-picrylhydrazyl (DPPH) assay method (Blois, 1958; Shishoo *et al*., 1999; Chhajed *et al*., 2007). To determine the free radical scavenging activity, a method based on the reduction of a methanolic solution of the coloured DPPH radical was used. To a set of test tubes containing methanol (3 mL), DPPH reagent (2 mg/mL) (50 μL) was added. The initial absorbance was measured. To these test tubes, methanolic solution of different test solutions (1 mg/mL) were added (10–50 μL). Ascorbic acid (0.5 mg/mL) was also added in the concentration of 10, 20, 30, 40, 50 and 100 μL. After 20 min, absorbance was recorded at 516 nm. The experiment was performed in triplicate. The percentage reduction in absorbance was calculated from the initial and final absorbance of each solution (Dhar and Taploo, 1982). The IC_50_ (nM/mL) values are shown in Table 1.

##### Superoxide anion radical scavenging effect

Measurement of superoxide anion scavenging activity of the synthesized compound was taken based on the method described by Nishimiki *et al*. (1972) and slightly modified. About 1 mL of nitroblue tetrazolium (NBT) solution (156 μM NBT in 100 mM phosphate buffer, pH 7.4), NADH solution (1 mL) (reduced form of β-nicotinamide adenine dinucleotide) (468 μM in 100 mM phosphate buffer, pH 7.4) and sample solution (0.1 mL) of compounds (10, 20, 30, 40, 50 and 100 μg) in distilled water were mixed and the reaction started by adding phenazine methosulphate (PMS) solution (100 μL) (60 μM PMS in 100 mM phosphate buffer, pH 7.4). The reaction mixture was incubated at 25 °C for 5 min, and the absorbance at 560 nm was measured against blank samples. Catechin was used as reference compound. All the experiments were performed in triplicate, and the results were averaged. The percentage of inhibition was determined by comparing the results of control and test samples. The IC_50_ (nM/mL) value are depicted in Table 1.

##### Nitric oxide radical scavenging effect

Nitric oxide generated from sodium nitroprusside in aqueous solution at physiological pH interacts with oxygen to produce nitrite ions, which were measured by the Griess reaction (Marcocci *et al*., 1994; Green *et al*., 1982). Scavenger of nitric oxide competes with oxygen leading to reduced production of nitric oxide (Mondal *et al*., 2006). The reaction mixture (3 mL) containing sodium nitroprusside (10 mM) in phosphate-buffered saline (PBS) and the compounds in different concentrations (10, 20, 30, 40, 50 and 100 μg) were incubated at 25 °C for 150 min. At every 30-min interval, the incubated sample (0.5 mL) was removed and Griess reagent (1 % sulphanilamide, 0.1 % naphthylethylene diamine dihydrochloride in 2 % H_3_PO_4_) (0.5 mL) was added. The absorbance of the chromophore formed was measured at 546 nm. All the analyses were performed in triplicate, and the results were averaged. The percentage inhibition of nitric oxide generated was measured by comparing the absorbance values of control and test. Curcumin was used as a reference compound. The IC_50_ (nM/mL) values are reported in Table 1.

#### In vitro antimitotic activity by *Allium cepa* (onion) meristem root model

Small bulbs (1.5–2.0 cm in diameter) of the common onion, *A. cepa* (2*n* = 16), were purchased from vendor at a local market. Prior to initiating the test, the outer scales of the bulbs and the dry bottom plate were removed without destroying the root primordia. The roots of *A. cepa* were grown in distilled water in Erlenmeyer flasks (200 mL capacity) under laboratory conditions (dark 24 °C). For each synthesized compound sample, after reaching a length of 3 cm (±0.5 cm), a series of six bulbs were placed in distilled water (pH 7.3) for 48 h and then onion roots were treated with the synthesized compound at 1 mg/mL concentrations of each tested compound. The test tubes were kept in an incubator at 22 ± 1 °C, and the test samples were changed daily at the same time. Several of the newly formed root tips were then cut from each bulb and examined for any visible morphological abnormalities. The bulbs with satisfactory root lengths (2–2.5 cm) were used in the study, while those with exceptionally long or short roots were discarded (on average 2–3 bulbs). Therefore, individual sets of five bulbs were used for each extract sample.

Distilled water (pH 7.3) was used as a negative control, and EMS (2 × 10^−2^ M) used as a positive control mutagen (Fiskesjo, 1993, 1997). After 24 h of exposure, several root tips were removed from the bulbs, fixed in 3:1 (v/v) ethanol (90 %)/glacial acetic acid (45 %) and stored overnight at 4 °C. The next day, they were placed in 70 % (v/v) aqueous alcohol and refrigerated until used. Allium roots were softened by digesting with HCl and rinsed the roots in water. After removing the water from the third rinse, the roots were covered with the orcein acetate stain. The roots were incubated in the stain for 12 min. During this time, the very tip of the root begins to turn red as the DNA stains the numerous small actively dividing cells at the tip. A root was transferred to the centre of a clean microscope slide, and a drop of water was added. Using a razor blade most of the unstained part of the root was cut off and discarded. The root tip was covered with a cover slip and then carefully pushed down on the cover slide with the wooden end of a dissecting probe. Care should be taken to push hard, but do not twist or push the cover slide sideways. The root tip should spread out to a diameter about 0.5–1 cm. Five slides were prepared per bulb.

##### Determination of cytotoxicity and genotoxicity

The following parameters were used for the determination of cytotoxicity and genotoxicity:

(i) the mitotic index (MI) was calculated as the ratio between the number of mitotic cells and the total number of cells scored and expressed as percentage using following formula as per standard procedures.
  $${\text{Mitotic}}\,{\text{index}} = \frac{{{\text{Number}}\,{\text{of}}\,{\text{dividing}}\,{\text{cells}}}}{{{\text{Total}}\,{\text{number}}\,{\text{of}}\,{\text{cells}}}} \times 100$$
(ii) Chromatin aberrations (stickiness, breaks and polar deviation) were used as end points for the determination of cytogenetic effects, and micronuclei (MNC) were scored in interphase cells per 1,000 cells (‰ MNC) (Freshney, 2000).
(iii) The most frequent abnormalities are shown in microphotographs. After 72 h of exposure to the test samples, the root lengths were measured and used as an index of general toxicity. The results for mitotic index and root length are expressed as percentage of the negative and positive controls. Visible morphological modifications, such as changes in root consistency and colour as well as the presence of swelling (c-tumours), hooks or twists in the roots, were also observed.

#### In vitro cytotoxicity activity by MTT assay method

##### Cell line and culture medium

The cancer cell line cultures of HEK 293 (epidermal kidney cell line), BT474 (breast cancer cell line) and NCI-H226 (lung cancer) were obtained from Pasteur Institute of India, Coonoor, India, and were cultured in RPMI-1640 and 10 % heat-activated New born calf serum with antibiotics [penicillin (1,000 I.U./mL), streptomycin (100 μg/mL) and amphotericin B (25 μg/mL)]. The cells were maintained at 37 °C in a humidified atmosphere with 5 % CO_2_ and were subcultured twice a week.

##### Determination of cytotoxicity by microculture tetrazolium (MTT) assay

The monolayer cell culture (100 μL) was trypsinized, and the cell count was adjusted to 3.0 × 10^5^ cells/mL using medium containing 10 % new born calf serum. To each well of the 96-well microtitre plate, the diluted cell suspension (approximately 10,000 cells) (0.1 mL) was added and kept for 24 h in incubator at 37 °C in 5 % CO_2_ atmosphere for cell monolayer formation. After 24 h, when a partial monolayer was formed at the bottom of the well, the supernatant was flicked off, the monolayer was washed once, and different drugs, i.e. synthesized compounds (100 μL), were added to the cells in microtitre plates. The plates were then incubated at 37 °C for 3 days in 5 % CO_2_ atmosphere, and microscopic examination was carried out and observations recorded every 24 h. After 72 h, the sample solution in the wells was flicked off; MTT dye (50 mL) was added to each well; plates were gently shaken and incubated for 4 h at 37 °C in 5 % CO_2_ incubator. The supernatant was removed and propanol (50 μL) was added; the plates were gently shaken to solubilize the formed formazan. The absorbance was measured using a microplate reader at a wavelength of 490 nm (Edmondson *et al*., 1988; Prasad *et al*., 2005; Chiruvella *et al*., 2008; Chang *et al*., 2007).
